# Post-Traumatic Stress Disorder (PTSD) and cardiovascular diseases: a systematic review and meta-analysis

**DOI:** 10.1192/j.eurpsy.2026.10241

**Published:** 2026-07-31

**Authors:** O. A. S. Al Nakhebi, V. R. Enatescu

**Affiliations:** Neuroscience, Victor Babes University, Timișoara, Romania

## Abstract

**Introduction:**

**Post-traumatic stress disorder** (**PTSD**) is a psychiatric condition characterized by intrusive memories, avoidance, negative mood alterations, and hyperarousal following trauma. Beyond its psychological burden, **PTSD** exerts multisystemic effects, including autonomic dysregulation, hypothalamic–pituitary–adrenal axis imbalance, inflammation, and health-risk behaviors, which increase cardiovascular disease (**CVD**) risk. **Conversely**, acute cardiac events can themselves precipitate **PTSD**, suggesting a bidirectional relationship.

**Objectives:**

This meta-analysis examined: (1) the risk of incident **CVD** among individuals with **PTSD**, and (2) the prevalence of **PTSD** in patients with **CVD**

**Methods:**

Following **PRISMA** guidelines, PubMed, Scopus, and Web of Science were searched up to May 2025. Eligible studies included observational or interventional human research reporting **CVD** incidence in **PTSD** or **PTSD** prevalence following **CVD**. Effect sizes were pooled using random-effects models. Heterogeneity was assessed with Q, I², and τ². Publication bias was evaluated with funnel plots and Egger’s test.

**Results:**

Twenty-nine studies (**n ≈ 2.7 million**) met inclusion. Eighteen assessed **CVD** risk in **PTSD** populations (n ≈ 2.6 million). Pooled analysis showed **PTSD** increased **CVD** risk by 75% (log RR = 0.56, 95% CI = 0.42–0.69; p < 0.0001). Eleven studies (n ≈ 148,000) reported **PTSD** prevalence following **CVD**, with pooled estimates indicating 25.3% (95% CI = 0.19–0.31) of cardiac patients met diagnostic criteria or exhibited clinically significant **PTSD** symptoms. Both directions showed substantial heterogeneity but consistent effects across study designs and populations.

**Image 1:**
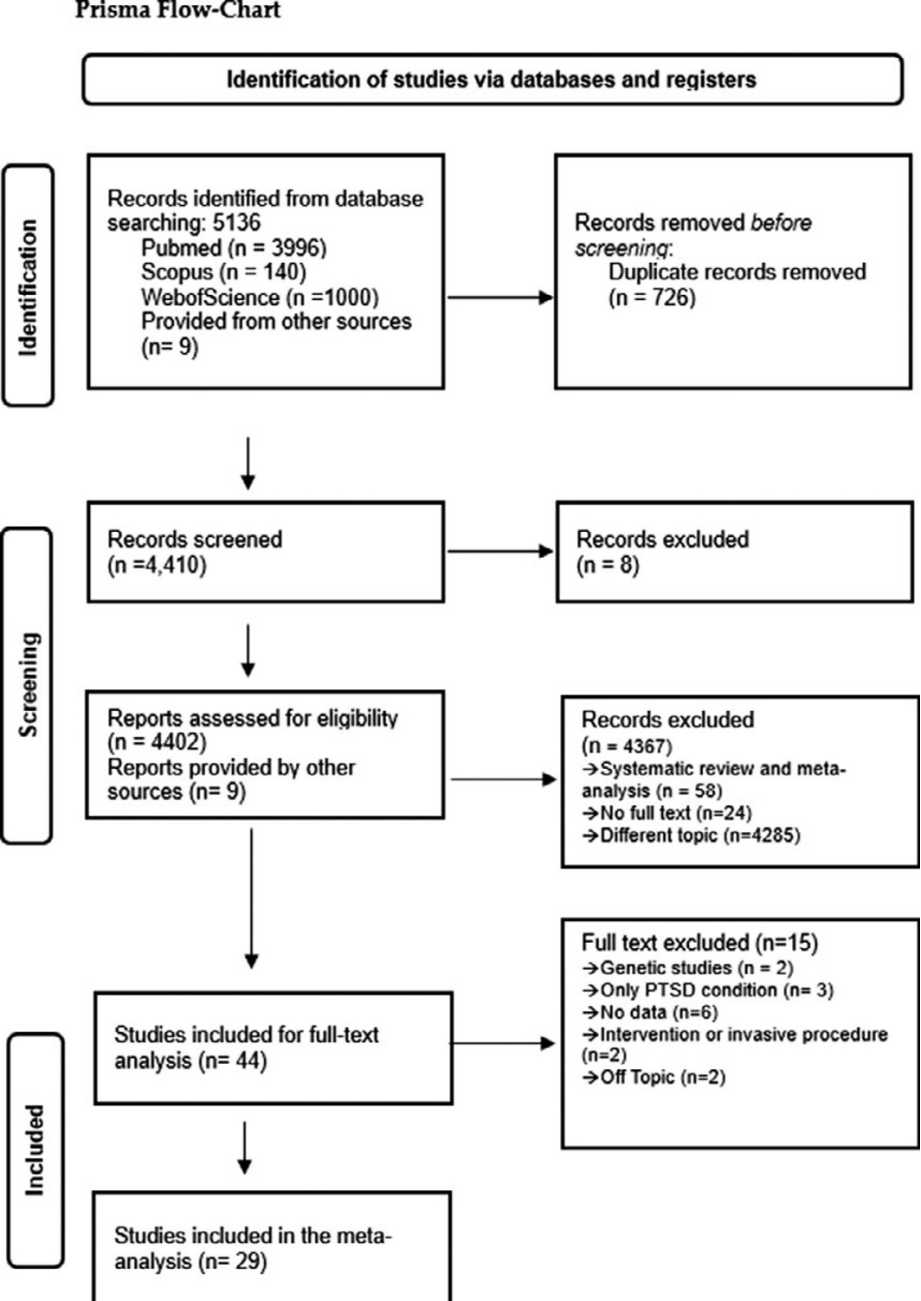
Image 1: Long description.

**Image 2:**
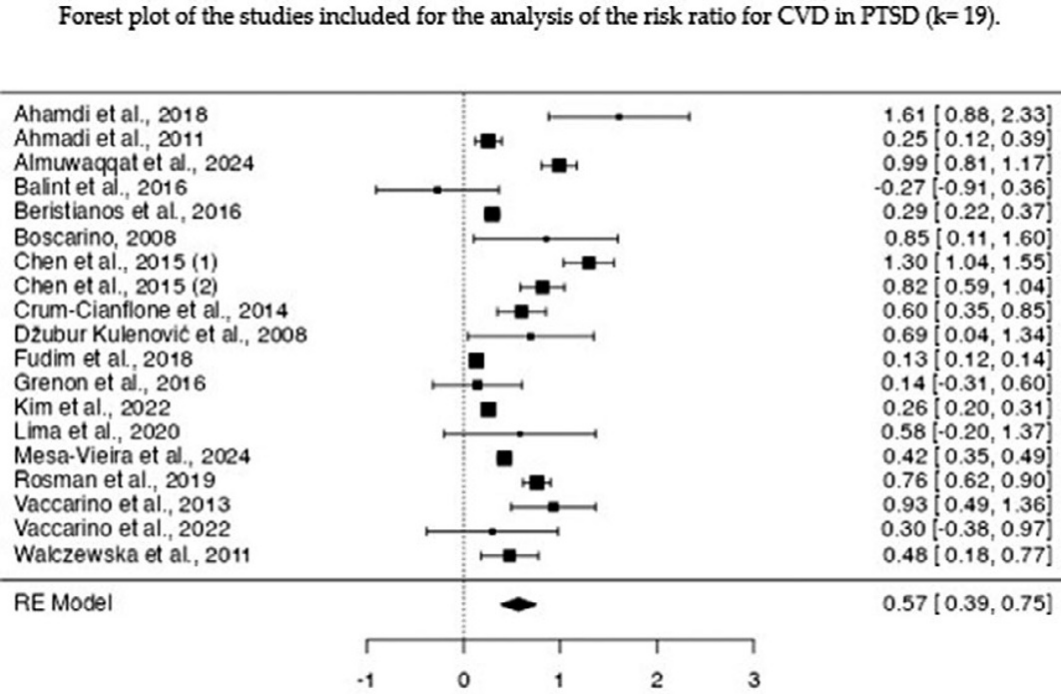
Image 2: Long description.

**Image 3:**
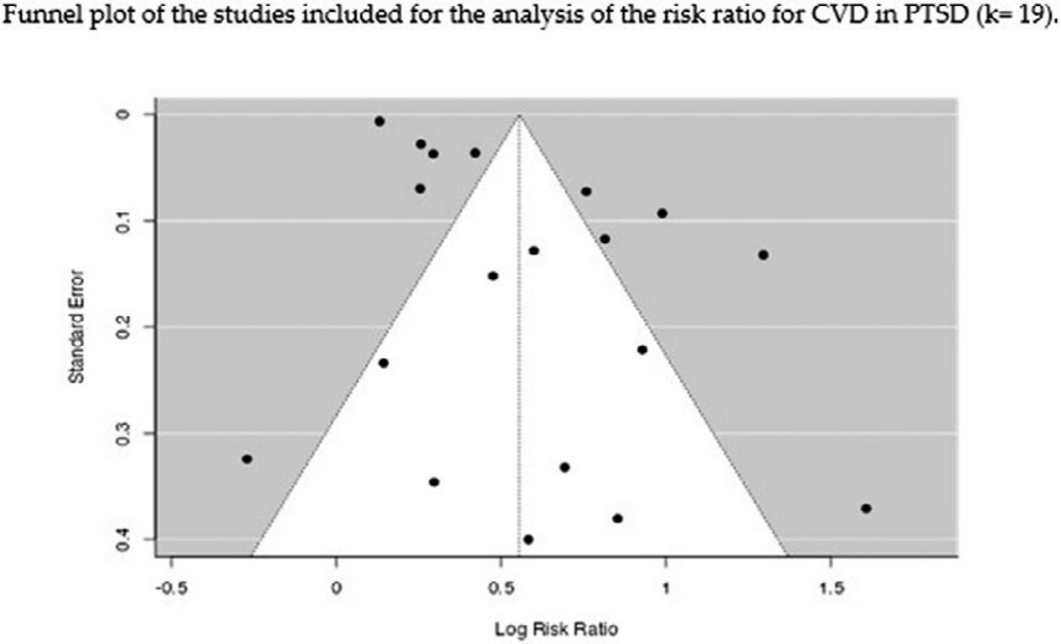
Image 3: Long description.

**Conclusions:**

**PTSD** and **CVD** are reciprocally linked. **PTSD** substantially elevates cardiovascular risk, while cardiovascular events act as potent psychological stressors, frequently triggering **PTSD**. These findings highlight the need for integrated care: trauma-informed strategies in cardiology and cardiovascular risk monitoring in psychiatric practice. Early identification and multidisciplinary interventions may help disrupt this pathological cycle and improve long-term prognosis.

**Disclosure of Interest:**

None Declared

## Long descriptions

### Image 1: Long description

The flowchart is divided into three vertical phases labeled on the left: Identification, Screening, and Included.

1. Identification Phase:

- Top box: Identification of studies via databases and registers.

- Left box: Records identified from database searching: 5136. This includes Pubmed (n = 3996), Scopus (n = 140), Web of Science (n = 1000), and Provided from other sources (n = 9).

- Right box (arrow from left): Records removed before screening: Duplicate records removed (n = 726).

2. Screening Phase:

- Central box: Records screened (n = 4,410).

- Right box (arrow from central): Records excluded (n = 8).

- Next central box: Reports assessed for eligibility (n = 4402) and Reports provided by other sources (n = 9).

- Right box (arrow from central): Records excluded (n = 4367). Reasons include Systematic review and meta-analysis (n = 58), No full text (n = 24), and Different topic (n = 4285).

3. Included Phase:

- Central box: Studies included for full-text analysis (n = 44).

- Right box (arrow from central): Full text excluded (n = 15). Reasons include Genetic studies (n = 2), Only P T S D condition (n = 3), No data (n = 6), Intervention or invasive procedure (n = 2), and Off Topic (n = 2).

- Final bottom box: Studies included in the meta-analysis (n = 29).

Navigate back to Image 1:.

### Image 2: Long description

The forest plot displays 19 studies on the left axis, a central plot area with a vertical dashed line at 0 representing the null effect, and numerical values on the right. The x-axis at the bottom ranges from -1 to 3.

Individual studies from top to bottom:

* Ahamdi et al., 2018: 1.61 [0.88, 2.33]

* Ahmadi et al., 2011: 0.25 [0.12, 0.39]

* Almuwaqqat et al., 2024: 0.99 [0.81, 1.17]

* Balint et al., 2016: -0.27 [-0.91, 0.36]

* Beristianos et al., 2016: 0.29 [0.22, 0.37]

* Boscarino, 2008: 0.85 [0.11, 1.60]

* Chen et al., 2015 (1): 1.30 [1.04, 1.55]

* Chen et al., 2015 (2): 0.82 [0.59, 1.04]

* Crum-Cianflone et al., 2014: 0.60 [0.35, 0.85]

* Dzubur Kulenovic et al., 2008: 0.69 [0.04, 1.34]

* Fudim et al., 2018: 0.13 [0.12, 0.14]

* Grenon et al., 2016: 0.14 [-0.31, 0.60]

* Kim et al., 2022: 0.26 [0.20, 0.31]

* Lima et al., 2020: 0.58 [-0.20, 1.37]

* Mesa-Vieira et al., 2024: 0.42 [0.35, 0.49]

* Rosman et al., 2019: 0.76 [0.62, 0.90]

* Vaccarino et al., 2013: 0.93 [0.49, 1.36]

* Vaccarino et al., 2022: 0.30 [-0.38, 0.97]

* Walczewska et al., 2011: 0.48 [0.18, 0.77]

At the bottom, the R E Model is represented by a black diamond centered at 0.57 with a confidence interval of [0.39, 0.75]. The majority of the study squares and their horizontal error bars lie to the right of the zero line, indicating an increased risk ratio.

Navigate back to Image 2:.

### Image 3: Long description

A scatter plot with a central white triangular funnel shape against a gray background.

* The horizontal X-axis is labeled Log Risk Ratio and ranges from negative 0.5 to 1.5.

* The vertical Y-axis is labeled Standard Error and ranges from 0 at the top to 0.4 at the bottom.

* A vertical dashed line representing the pooled effect size is positioned at approximately 0.55 on the X-axis, forming the apex of the funnel at Y equals 0.

* The data consists of 19 black circular points representing individual studies.

* Distribution: Most studies are clustered near the top of the plot between 0 and 0.1 Standard Error.

* Symmetry: The points are distributed on both sides of the central dashed line.

* Outliers: There are several points outside the white funnel area in the gray shaded regions, including one at approximately X equals negative 0.3, Y equals 0.32, and another at X equals 1.6, Y equals 0.37.

Navigate back to Image 3:.

